# Research on ethics in two large Human Biomonitoring projects ECNIS and NewGeneris: a bottom up approach

**DOI:** 10.1186/1476-069X-7-S1-S7

**Published:** 2008-06-05

**Authors:** Birgit Dumez, Karel Van Damme, Ludwine Casteleyn

**Affiliations:** 1 Center for Human Genetics, Department of Medicine, Katholieke Universiteit Leuven, Herestraat 49, 3000 Leuven, Belgium

## Abstract

Assessment of ethical aspects and authorization by ethics committees have become a major constraint for health research including human subjects. Ethical reference values often are extrapolated from clinical settings, where emphasis lies on decisional autonomy and protection of individual's privacy. The question rises if this set of values used in clinical research can be considered as relevant references for HBM research, which is at the basis of public health surveillance. Current and future research activities using human biomarkers are facing new challenges and expectancies on sensitive socio-ethical issues. Reflection is needed on the necessity to balance individual rights against public interest. In addition, many HBM research programs require international collaboration. Domestic legislation is not always easily applicable in international projects. Also, there seem to be considerable inconsistencies in ethical assessments of similar research activities between different countries and even within one country. All this is causing delay and putting the researcher in situations in which it is unclear how to act in accordance with necessary legal requirements. Therefore, analysis of ethical practices and their consequences for HBM research is needed.

This analysis will be performed by a bottom-up approach, based on a methodology for comparative analysis of determinants in ethical reasoning, allowing taking into account different social, cultural, political and historical traditions, in view of safeguarding common EU values. Based on information collected in real life complexity, paradigm cases and virtual case scenarios will be developed and discussed with relevant stakeholders to openly discuss possible obstacles and to identify options for improvement in regulation. The material collected will allow developing an ethical framework which may constitute the basis for a more harmonized and consistent socio-ethical and legal approach. This will not only increase the possibilities for comparison between data generated but may also allow for more equality in the protection of the rights of European citizens and establish trustful relationships between science and society, based on firmly rooted ethical values within the EU legislative framework.

These considerations outline part of the research on legal, socio-ethical and communication aspects of HBM within the scope of ECNIS (NoE) and NewGeneris (IP).

## Background

Human biomonitoring (HBM) is a useful tool to assess human exposures to environmental agents and their health effects, based on sampling and analysis of an individual's tissue and fluid. Biomarkers indicate steps in a series of events leading to diseases that may result from exposure to (toxic) pollutants or harmful agents. Because many significant diseases develop over longer periods of time, methods for detecting early markers that can predict risk are important for disease prevention.

Current and future research activities using human biomarkers are facing new challenges and expectancies on sensitive socio-ethical issues, especially in the environmental public health context where activities should lead to policy, preventive actions and raising awareness at population level. Hence the notion of public interest comes more and more to the forefront, leading to substantial differences as compared to clinical settings, in terms of risk-benefit balance and consequences for the individual and for the entire population and in terms of required communication practices. Reflection is needed on the ways to balance individual decision-making as an expression of individual rights against the public interest. HBM studies often require a maximal participation of given populations in order to provide reliable knowledge, which may then be beneficial for protecting people through preventive action. The appropriateness of the current legal and ethical framework, mainly issuing from and applicable to clinical medicine, and therefore representing a strongly individual-oriented approach, should therefore be assessed. At the same time, domestic legislation is not always easily applicable in international collaborative research projects, causing delay and putting the researcher in situations in which it is unclear how to act in accordance with all necessary legal requirements on ethical aspects of research. As a consequence, scientific opportunities may be missed and important developments of which many individuals will benefit, hampered.

The key questions at stake are how research subjects involved in HBM research can be adequately and equally protected throughout the whole of Europe whilst at the same time scientific research in the field of environmental health developing human biomarkers and also surveillance studies and practices using HBM with a view to protect people, are not simply being restricted nor prevented for the wrong reasons. Documenting and analyzing current practices is a method to allow identification of both bottlenecks and not allowable shortcuts in this process. These practices can be represented and discussed as such or translated into virtual cases. The latter may facilitate open discussions.

Indeed, whilst in general there is a willingness to be in compliance with what can reasonably be expected from ethically correct conducted research, researchers are faced with a labyrinth of rules and guidelines, often open for interpretation, which leaves them worried about the fact that the legitimacy of the research which is ongoing might be challenged [1]. In what follows, specific ethical challenges in HBM studies will be highlighted trough such cases. They relate to decision-making processes, including informed consent, to communication aspects, and to ethical assessment and approval procedures. The methodology of the ethical research within ECNIS  and NewGeneris  projects and the search for intermediate solutions to practical problems that researchers within the group are faced with will be explained briefly.

## Current practices

The World Wildlife Fund (WWF) has organised several HBM surveys in the past few years testing people across the whole of Europe . The goal of these surveys was not to prove a scientific hypothesis, but to raise awareness of the general public about the extent of chemical pollution in Europe and to show how essential a strong European chemical regulation is. The results have shown that persons tested are 'contaminated' with a cocktail of persistent, bio-accumulative and toxic man-made chemicals. Although the study was conducted in full respect of the rights of each participant, questions were brought up about the exact procedures to follow, on which it seemed difficult to get consistent answers. According to the protocol of the Convention on Human Rights and Biomedicine (ref [2], article 7), every "research" project has to be submitted to an independent research ethics committee and approval of an ethics committee has to be required before the start of the project. In the case of WWF the question was raised weather such a campaign had to be considered a "research" project or not, since its purpose is not primarily to produce knowledge but to raise awareness. Furthermore, only very few samples were collected in a large number of countries (13 families from 12 EU countries)  and it was unclear whether ethical approvals were required in each country.

Other difficulties in the correct implementation of the rules and guidelines relate to the secondary use of samples and/or personal data. According to the EU Privacy legislation [3], data should not be processed further in a way incompatible with the specific purposes for which the data have been collected. However, further processing of personal data (the so called secondary use of data) is generally not considered incompatible with the purposes for which the data have previously been collected provided that Member States furnish suitable safeguards (Article 13 of the Directive 95/46/EC). If a new purpose is found incompatible, the research proposal is considered as a new project and consequently a new individual informed consent must be requested. There may be exceptions: if the provision of information to the research subject proves impossible or would involve disproportionate efforts, a new consent is not necessary on the condition that this has been explained to the national privacy authority where the notification is done. Divergent interpretations on the "compatibility of a purpose" and on what is "impossible or disproportionate effort" reflect uncertainties about the best interpretation of the Directive and may cause confusion. Can, for instance, in the development of new and validating DNA repair phenotypes for which an informed consent was previously obtained for genotyping, the samples be reused without obtaining new individual consent for phenotyping?

The above examples illustrate the need to clarify and maybe even to facilitate formal legal aspects and ethical constraints of HBM practices. This need is not only present for academic research, but also in other activities using HBM, such as surveillance programs and raising awareness campaigns.

Specific for many studies in environmental health is the direct involvement of various stakeholders from the start on: industry, NGO's, politicians and authorities. A Eurobarometer survey published by the European Commission showed that medical and health organizations such as 'The Red Cross' and 'Médecins sans frontières' are the most trusted sources of health information across the EU (trusted by 84%). Consumer organizations are the second most trusted source (67%), closely followed by schools and universities (65.5%) and environmental organizations (63%). The media was trusted by 39% of people surveyed, governments by 23% while business and political parties had the lowest trust rating when it comes to health information (16% and 11% respectively) .

However, in HBM studies the latter are playing a major role besides the health professionals. If for instance an increase in lung cancer risk has been attributed to historical cadmium pollution due to industrial activities in a certain region, the population concerned will be deeply alarmed about the impact of the pollution on their health. They will look for specific actions from the policy makers and the industry at the origin of the pollution and for information on individual health risk. According to the "polluter pays" principle, the industry at the origin of the pollution should contribute to sanitation of the contaminated soil and to evaluation of the effects of their interventions. A HBM study in the affected area may be one of the tools. However, in this kind of situation, how will the (affected) population perceive a (financial) involvement of industry in the HBM study? Will the people have trust in a transparent and fair interpretation and communication of data?

In many cases, communication of results to the research subjects is a difficult endeavor, as both the right to know and the right not to know need to be respected. The information provided should be correct and understandable and avoid raising unnecessary alarm, although interpretation of the data collected may be difficult and not straightforward, as may be illustrated by the following example.

Under the auspices of the United Nations Environment Program (UNEP), at the ratification of the Stockholm Convention on persistent organic pollutants (POP's) in 2004, it was decided to perform a reduction of the twelve most present POP's in the environment. To test the efficiency of the policy agreements, the World Health Organization (WHO) coordinates currently the fourth survey round on pollutants in breast milk throughout the world in order to measure the levels of POP's, such as PCBs, PCDDs and PCDFs . The results demonstrate that breast milk contains many pollutants. It is know that during breastfeeding, a transfer of the body burden from mother to child takes place. Communication of such results to the mothers risks to raise panic and induce anxiety and even guilt feelings. On the other hand, it is scientifically clear that breast-feeding has many advantages to babies and mothers with respect to protection against diseases and to the comfort and physical closeness it entails. The foremost question to pose is how not to overestimate the risks or downplay the benefits of breast-feeding to the mothers? Three possibilities in communication of results can be considered. The first one would be never to communicate on individual level as the general rule. Communication of results will then only include information at the collective level and focus for example in the specific situation of the downward trend in the concentration of POP's over time. However research participants have a right to know the data that have been processed, as guaranteed by the EU Directive 95/46/EC (article 12 of [3]). Therefore communication of individual results should be performed either as a general rule, unless specified otherwise by the study participant during the informed consent procedure, either on request only.

The right to know or not to know may also entail close relatives. Consider the case where employees in a factory are long-term exposed to pollutants that might impact their health status and the health status of their offspring. Since there exists a growing trend in society to lift conflicts of various natures at judicial level, it would not be unrealistic to be confronted with the right to know effected by the children of exposed employees who require compensation for possible damage on their health and lives.

As stated above, the protocol of the Convention on Human Rights and Biomedicine indicates that biomedical research can only be conducted with the approval from a research ethics committee. The lack of harmonization of ethics committee processes across Europe, the need for a simplified research governance framework, and the "inconsistency" as a result of the inherent variability in moral judgment were reported [4].

Also the composition of ethics committees across Europe varies [5]. Risk and benefit analysis in environmental health studies may require a specific competence that is not always available in REC's. An ethics committee with roots solely in the clinical world may have a very different appreciation than an ethics committee which has more expertise in public health matters. For instance the use of a tumor marker (TM) to screen for cancer needs a different judgment then the use of the same TM to assess exposure to pollutants. A marker might be acceptable in a clinical research study in view of identifying early markers of disease. Using the same TM to assess impact of exposure at population level however is a different matter. Although in the latter case it is not the objective to identify at risk persons, results will become available that give indications for an elevated risk at individual level. This is especially difficult if the TM is not highly predictive, implying that, as a consequence of large scale testing, a considerable amount of false positive persons will be revealed. These persons may go for diagnostic procedures that may cause unnecessary side effects, not to forget that the cancer treatment itself may cause a lot of side effects, even if the test may detect insignificant tumors that would never become clinically life threatening. From a medical point of view, it is not yet known if the test actually saves lives and if the benefits of screening outweigh the risks of follow up diagnostic tests (e.g. biopsy) and cancer treatments. With respect to its relevance for environmental exposure, it is not generally accepted nor validated that a specific causal linkage exists. Being given this information, the issue is whether the use of this TM test in environment and health survey settings would be acceptable? If it is acceptable, which are the conditions? Who decides on the use and on the acceptability of using the TM test? Should the decision be taken by scientists and/or by policy makers and/or by a local ethics committee? A clinically oriented ethics committee which is not familiar with the reasoning of public health practices may content itself with approving the project insisting mainly on the free informed consent.

At recruitment phase an informed consent procedure is a necessary condition to involve healthy volunteers in HBM research in all EU Member States. However, procedures and contents of the process vary significantly across EU Member States. Particularly difficult is the involvement of children. A variety of large mother-child cohorts are set or being set up in many European countries . Increasing awareness of long-term health effects from early life towards childhood and adolescent age is the basis of this approach. Knowledge on the genotoxicity of contaminants in children is a necessary scientific basis for possible further European regulatory activities. Who will decide on the participation of children in such studies? The acceptability of risks that the research may impose on the children and the respect for their autonomy are considered relevant ethical considerations. However, questions remain on the age that children are able to make decisions consciously and independently [5]. In some European Member States collection of personal data and/or samples of children related to medical and health care purposes are kept under auspices of the parents until the children reach the age of eighteen. Curiously, at the same time, in the case where databases keep data and/or samples of young children related to criminal purposes, the children have the authority to decide for themselves from the age of thirteen. How come in one case children are assumed to be able to make autonomous decisions and in another case not? Biological age is certainly not the only criterion to decide upon [6].

Another challenge emerges in many EU Member States: the inclusion of many ethnic and cultural minorities in research projects. In research on gene-environment interactions, there is a growing interest to integrate different ethnic groups, since they represent different dietary habits and possibly different exposures. Imagine that mothers are being recruited to participate in a large mother-child cohort. Different ethnicities or religious backgrounds might stand for considerable differences in the extent to which the mother makes her own decisions or otherwise the father decides for her, whether or not she may participate in the study cohort. In an increasingly pluralistic society, how should culture and identity be respected but at the same time the autonomy and dignity of the mother guaranteed without hampering the dignity of the father [7]?

### Ethics, an obstacle for research?

In HBM research ethics often comes across at first in the legal obligation to comply with all national and EU legislation, irrespective of the 'thicker' meaning of conducting science in an ethical way. For instance, the legal requirement to apply to local ethics committees for approval before the start of research may be perceived as a bureaucratic "nuisance" because it stands for a bunch of time consuming paper work that might cause delay in research. Moreover, in the existing ethical framework including national, European and international regulations, international conventions and declarations, as well as guidelines and opinions (*i.a. *EU Directive 95/46/EC, Council of Europe's Convention on Human Rights and Biomedicine), researchers may be put in situations in which it is unclear how to act in accordance with all necessary legal requirements of ethical aspects of research. In transnational research projects, which are key tools in further investigations of the health impact caused by environmental factors on a large scale, and in which transfer of sensitive personal data and/or biological samples from one Member State to another is a common practice, the labyrinth of rules and guidelines becomes an even larger clew. As a consequence, significant scientific developments may be missed whilst juggling with ethical concepts and rules, which in the worst of cases may even overlook the main objective. Alternatively, ethical approval might be shortcut.

### Decision making processes

Analysis of decision making processes are at the core of the below presented approach and in line with the methodology for comparative analysis of the determinants of ethical reasoning. Any situation calls for the one foremost question to be analyzed: who decides upon what for whom, why, how, on which grounds and with which consequences for whom? Decision making processes are to be considered at several levels. Who initiates the study? Who decides on the design of a research project? Who decides on the use of a certain biomarker? Why does a healthy volunteer decide to participate in it?

In environmental health research, it may be unclear whether the concept of autonomy, as implemented via informed consent, is always the most appropriate or the necessary way for decision making in a research domain where societal interests often directly surpass the individual interest. An additional question in that respect is whether, and to what extent-also in clinical situations- an informed consent can be considered free and authentic, and based on a correct understanding of the issue at stake. Some of the above examples illustrate the complexity of finding the appropriate way to implement the informed consent principle and how it should apply.

It is obvious that a signed consent on paper alone does not adequately protect the rights and dignity of every participating individual. By applying ethics in a procedural manner, the real meaning of informed consent – to make well informed and autonomous decision – may be jeopardized.

Anyhow, also when individual informed consent would not be the key regulatory principle in some HBM research or surveillance practices, it is important that the person should be fully informed on what the research or practice is about, and on the reasons why no consent is asked for.

### Ethics committees

Often the ethics committee has a unique strong position in the decision making process on the acceptability of a project. However, the composition of members of local ethics committees is often bound for decision making in clinical situations.

Questions related to the role of local ethics committees are relevant since the incidence of *ad hoc *and very local debates indicate that there is little consistency, which may even lead to a 'shopping' phenomenon: studies will be undertaken where the 'resistance' is low.

### A need for adapted communication strategies?

As refered to above, according to the EU Privacy Directive every research subject has the right to know about all personal data that has been processed about him or her, including individual results of research. At the same time also the right not to know is preserved [3]. In many HBM studies individual results are often not provided to the research subjects based on one of the following arguments: (1) the lack of relevance of the results at individual level; (2) too limited time and/or resources; and (3) fear of causing (unnecessary) alarm; (4) scientific uncertainty; (5) lack of potential for remediation. In fact, the currently used biomarkers may be measured without the possibility for implicit information to the research subjects, to the general public or to other authorities on the potential health consequences or on measures for policy development and prevention. Although individual results are often not communicated since they may not (yet) be meaningful at individual level, the research subjects have a legal right to know their individual results of research.

Furthermore, the question emerges what to do in case of conflict between the right not to know and the duty to inform? During research, results at individual level can be found that need preventive or curative action. However, prior to the commencement of research, the wish of the participant to know or not to know has to be established. Although research situated in the public health context is generally carried out without presumed direct benefits for the individual, how can be guaranteed that the relevant individual receives appropriate preventive action or therapy?

## Methodology for critical analysis of ethico-legal contexts of HBM practices

The need for 'rethinking' the current ethical framework is studied using a methodology for assessment of consistency in respect to ethical values generally considered as part of EU socio-ethical discourse. This methodology has been developed in the context of three earlier European projects [8-11]. Application of biomonitoring in the workplace analyzed with the same method has led to the development of appropriate rules to give guidance on how ethical and responsible HBM is consequently undertaken. It is expected that these lessons learned might be supportive to HBM practices in the area of public health. Four successive steps can be distinguished: (1) identification of the global socio-economical and political context, (2) analysis of the *de facto *practices, (3) analysis of the national decision making process and (4) evaluation against European standard values highlighting respect for human dignity, social justice, solidarity and democratic participation.

A bottom up approach is adopted, meaning that continuous investigation and analysis of current research practices is performed, starting with the information collected from formal and informal contacts, interviews, questionnaires etc. Paradigm cases are selected and virtual case scenarios developed which will constitute a template for systematic discussion on the issues of concern with all involved parties. The virtual case scenarios start from real cases, with additional 'elements' that facilitate focusing on these issues. This approach not only allows collecting precise information on real life complexity and obstacles, but also inspires the development of solutions towards a consistent ethical approach together with all involved parties. With the methodology -that was proven efficient in previous research- serving as a solid backbone, the reflection on the paradigm cases and other (virtual) case scenarios, conducted in an open atmosphere by diverse stakeholders, creates the opportunity to assess not only whether there is a justified need for rethinking the ethical framework for environmental health research, but also to identify possible solutions. The method also offers insight into differences in practices and in perception on the issues at stake in different Member States and into the rationale for these differences. Consequently, the analytical comparison of practices taking into account specific (national) contexts may lead to the identification of levers for a harmonized approach in terms of respect of well-defined common values, but without necessarily making the practices identical.

### An intermediate problem solving approach

Working with different research teams in the EU funded projects ECNIS and NewGeneris learned that the focus of activities with respect to ethical analysis had to be widened to fulfill also more immediate and practical needs. The challenge consists of elaborating very concrete and clear practical guidelines and solutions that not only guarantee the respect of ethical values, but at the same time reduce the perceived burden of ethical constraints, both for the research subjects and the researchers.

From the cases collected, it became apparent that due to the specific public health context, there is a need for appropriate communication strategies, at recruitment, during and also at the end of a project. Appropriate communication strategies are being developed for biomarkers of exposure, effects, risk and susceptibility. For each category of biomarkers possible ethical pitfalls are identified.

In the labyrinth of rules and guidelines, a proposal for solution regarding secondary use, transfer of data and the need for ethical approvals and notification in transnational research consists out of two levels: proposals for facilitation of international research in the context of environmental and public health are being developed and tested at the project level and will later on result in a proposal at European level. The proposal at project level encloses in the first place a very practical roadmap showing how to comply with legislation, and is then followed by a proposal for facilitation, representing possible situations the researcher might find himself in with corresponding legal requirements.

## Conclusion

Current and future research activities using human biomarkers are facing new challenges and expectancies on sensitive socio-ethical issues. Profound rethinking of the current ethical and legal framework, issuing from clinical medicine, putting emphasis on decisional autonomy, is desired since it does not seem to give appropriate guidance when the notion of public health moves to the forefront. Specific bottlenecks relate, amongst others, to communication of results – which are often open for interpretation – at individual, as well as at collective level, translation into policy, and secondary use of material in transnational research. The need for research on legal, socio-ethical and communication aspects of HBM to tackle these bottlenecks is fully recognized and being carried out within the scope of several EU funded projects, such as ECNIS NoE and NewGeneris IP, striving to contribute to solutions in which the human rights and dignity of research subjects are protected, whilst simultaneously promoting development and use of human biomarkers. Research is performed by a bottom-up approach, based on a methodology for comparative analysis of determinants in ethical reasoning, allowing to take into account different social, cultural, political and historical traditions, in view of safeguarding common EU values. Based on the information collected in the field, paradigm cases and virtual case scenarios are developed. These are discussed considering relevant stakeholders in order to openly discuss possible obstacles and to identify options for improvement in regulations.

A harmonized socio-ethical and legal approach, including procedures for effective and appropriate communication, not only increases the possibilities for comparison between data generated but may also allow for improved equality in the protection of the rights of citizens of Europe and to establish trustful relationships between science and society, based on firmly rooted ethical values in EU legislative framework, which is crucial for further developments within the public and academic researchers.

## Competing interests

'The authors declare that they have no competing interests.

## Authors' contributions

All authors participated in the design and performance of the study. All authors read and approved the final manuscript.
